# Exploring the beliefs and attitudes of private general practitioners towards national health insurance in Cape Town, South Africa

**DOI:** 10.4102/phcfm.v11i1.2189

**Published:** 2019-10-17

**Authors:** Sheena Mathew, Robert Mash

**Affiliations:** 1 Division of Family Medicine and Primary Care, Faculty of Medicine and Health Sciences, Stellenbosch University, Cape Town, South Africa

**Keywords:** national health insurance, general practice physicians, primary care, family practice, private practice

## Abstract

**Background:**

Private general practitioner (GP) participation in the national health insurance (NHI) is necessary to address doctor shortages and achieve universal health coverage. An in-depth understanding of GP’s views on the NHI is needed to inform implementation strategies.

**Aim:**

To explore the beliefs and attitudes of GPs towards the proposed NHI system.

**Setting:**

Cape Town, South Africa.

**Methods:**

This was a descriptive, exploratory, qualitative study using semi-structured interviews. Eleven GPs were recruited using purposeful snowball sampling from different practices and communities. Thematic data analysis was conducted using the framework approach and Atlas.ti software.

**Results:**

Although GPs saw the need for NHI, they felt that the government was antagonistic towards the private sector and had not engaged in a dialogue. They were wary of integration into a nurse-led primary care system and of being coerced. They felt that the public sector lacked the necessary financial and administrative capacity, and were concerned about the level, efficiency and sustainability of reimbursement, and the criteria to be used to accredit practices. General practitioners anticipated that the NHI would favour multidisciplinary teams and group practices. They also had mixed ideas about the impact on practice with some expecting higher workloads, stress and costs with reduced quality of care, while others saw more comprehensive care, better incomes and increased patient satisfaction.

**Conclusions:**

While GPs are essential for the success of the NHI, there are many concerns regarding government policy, plans for implementation and the consequences for GP practice. Many of the concerns expressed could be tackled by greater policy dialogue and clarification.

## Background

In 2005, all World Health Organization (WHO) member states made a commitment towards achieving universal health coverage.^1^ It was a collective expression of belief that people should have access to quality health services without risk of financial ruin or impoverishment.^2^ This expansion of health coverage would result in lower child and adult mortality, with the beneficial effect on child mortality being the maximum in poorer countries.^3^

Research plays a vital role in generating evidence to reform health systems in order to achieve universal health coverage.^2,4^ The different political environments, cultures and inherited legacies of countries have resulted in diverse reforms,^5^ nevertheless evidence suggests that the following reforms are effective: shifting high hospital spending towards primary care, increasing government expenditure on primary care and introducing public–private partnerships to expand health coverage.^6,7^

In Africa, countries such as Ghana, Nigeria, Rwanda, Kenya and Mali have commenced with national health insurance (NHI) as a key strategy. Health reforms in these countries have led to increased government spending on health, with taxation as a key source of revenue; increased coverage of primary health care; and decreased out-of-pocket spending.^5^

The introduction of the NHI is also a key strategy in South Africa in response to the global call for universal health coverage.^8^ The current two-tiered health care system comprises an under-resourced public health sector serving over 80% of the population and an over-resourced private health sector serving less than 20% of the population. This has contributed to the inequity in our current system, in addition to being unsustainable, costly and hospital-centric.^9^ South Africans are dissatisfied with both the private and public health sectors and are ready for health reforms.^10^

In 2011, the government launched the NHI Green Paper, outlining a phased project over 15 years to provide improved access to quality health services for all citizens, irrespective of their financial circumstances.^9^ One of the public–private partnership activities in the NHI pilot sites during the early phase of improving the quality of public sector primary care has been to contract private general practitioners (GP) to provide services in primary care facilities.^11^ The uptake by GPs was initially low owing to the poor remuneration offered. General practitioners were asked to support nurse-led primary care clinics. This approach is not the same as that envisaged in the NHI whereby GP practices could be accredited to provide a package of primary care to a registered practice population. The exact nature of this package has not yet been defined.

Private sector participation is vital for the effective implementation of the NHI as more than half of the 8.8% of gross domestic product spent on health is invested here.^12^ The importance of the private sector is further illustrated by the extent of their human resources and infrastructure.^12^ The South African Private Practitioner Forum’s submissions on the NHI maintain that reforms should only be pursued if they are practically implementable and affordable. The forum also expressed concerns over the negative portrayal of the private sector.^13^ The resolution of these concerns is necessary in order to ensure private sector participation in the implementation of the NHI.

Public–private collaborations can play an important role in alleviating resource and personnel shortages that affect the public sector.^7,14^ Addressing private GPs’ concerns regarding the government’s capability to manage the new system, over-utilisation of services and reimbursement fears^15^ will promote better engagement between the private sector and the government.

Further research within this sector is required in order to provide guidance regarding the implementation of policies and the best ways of engaging with the private sector. This study looked to address this gap in our current knowledge regarding the views of private GPs 5 years after the initial Green Paper was introduced. The study aimed to explore the beliefs and attitudes of private GPs in the Cape Town metropole towards the proposed NHI system.

## Methods

### Study design

This qualitative study used a descriptive thematic analysis approach with semi-structured interviews to explore the beliefs and attitudes of GPs towards the NHI based on their experience of general practice and the initial steps to implement the NHI in the South African health system.

### Study setting

The study was conducted among private GPs in the Cape Town metropole, Western Cape. Cape Town has a population of 3.74 million, making it the second most populated city in South Africa.^16^ The leading causes of death in the Western Cape are cardiovascular disease (25%), malignant neoplasm (16%), infectious and parasitic diseases (10%), injuries (9.7%) and HIV/AIDS (8.4%).^17^ Non-communicable diseases account for 58% of deaths in the Western Cape, compared to 38% nationally.^17^ According to a private sector database, there are 2641 GPs and 2193 practices based in the Western Cape.^18^ Private GPs may serve affluent communities, where the majority are fully insured, as well as poor communities where many patients will be fee-for-service users or part of managed care or hospital insurance packages. General practitioners might be in solo or group practice and might manage their own business or work for a private health service provider.

At the time of this study, the NHI was outlined in broad terms in a White Paper.^19^ The proposal was that the NHI scheme would cover all South Africans and provide universal health coverage for a package of health care services. There would be a split between the purchaser of health services and the providers of health care services. The purchaser would accredit providers in either the public or the private sector who could provide the package of care. Remuneration of these providers would primarily be based on a registered practice population and capitation. Implementation of the NHI would most likely start with primary health care and the package would be based on the services offered by a nurse-led primary care clinic with access to a doctor. All working South Africans would be obligated to contribute to the NHI from their salaries, but would also have the option to pay for additional medical insurance in the private sector. It was envisaged that the NHI would be implemented progressively over a 15-year period and that in the first 5 years the focus would be on improving the quality of care in the public sector.^11^ Key initiatives were the establishment of the Office for Health Standards Compliance, primary health care re-engineering, and improvements in the infrastructure of public sector facilities. During this period, NHI pilot districts were created in each province and within these pilot districts private GPs were approached to contract with the department of health to support primary care clinics.

### Selection of participants

Practicing GPs who held leadership roles within GP organisations, such as the South African Medical Association, Qualicare, South African Academy of Family Physicians, Emerging Market Healthcare and Medicross, were selected for their awareness of or involvement in the NHI processes. These individuals were then asked to nominate other prominent GPs (snowball sampling) who might have strong opinions on the topic or who could represent the point of view of different types of GPs. Snowball sampling intended to select GPs from practices that were largely fee-for-service, with patients from low socioeconomic groups, to practices that had substantial numbers on managed care insurance packages, with patients from middle socioeconomic groups, to those that were largely medical aid based in high socioeconomic groups. A mix of solo and group practices as well as GPs that worked in both the private and the public sectors were also selected. Fifteen GPs were initially contacted telephonically for their participation in the study, and interviews were stopped when no new themes emerged.

### Data collection

Individual, face-to-face interviews were conducted in English using an interview guide during 2016. The interviews were held at the GP’s practice, audio-recorded and lasted up to 60 min. The interview guide explored their beliefs and attitudes towards the NHI, the proposed implementation process and the implications for their practice. Beliefs were seen as ideas that the interviewee held to be true about the NHI, while attitudes were seen as their mental disposition towards its implementation and implications for practice. The interview guide was developed based on key areas outlined in the government’s NHI green paper^8^ and previous issues highlighted in the literature.^13,14,15^ The interview guide was reviewed by two experts for content and construct prior to use. The interview guide was piloted to ensure that the data generated were valid for the research purpose. The guide contained a number of open questions that could be used to explore GP’s beliefs and attitudes towards the NHI, its implementation and impact on general practice. The opening question was, ‘What do you think about the government’s proposed NHI?’. The research supervisor reviewed the pilot interview recordings to ensure their quality and also interviewed the principal researcher to enable reflexivity and self-awareness of viewpoints that might influence the interview or analysis. The principal researcher’s previous work experience as a GP and current position as a family medicine registrar within the public sector gave her an insight into both sectors.

### Data analysis

Field notes were used to document information pertinent to the practice setting. Each audio recording was transcribed verbatim and checked for errors or omissions. The framework method supported by Atlas.ti software was used to analyse the data. This entailed:

- Familiarisation with the transcripts.
- Creating an index of codes that emerged from the data, which were organised into categories aligned with the study objectives.
- This thematic index was then used to code all the data. The coding consistency was checked by the research supervisor after the initial few interviews and once sufficient consistency was achieved, the coding rules were applied to all the transcripts.
- Charts were used to bring together all the data with the same code.
- These charts were then used to interpret the data and extract themes. The final analysis involved exploring the nature and range of opinions within a specific theme as well as the relationship between themes.

### Ethical consideration

This study was approved by the Health Research Ethics Committee of Stellenbosch university (Reference: S15/07/160). This committee abides by the ethical norms and principles of research established by the Declaration of Helsinki, the South African Medical Research Council Guidelines as well as the Guidelines for Ethical research: Principles Structures and Processes 2004. All participants gave informed consent.

## Results

Overall, 15 GPs were contacted, and 11 were willing to participate. Among the GPs who did not participate, three GPs declined owing to time constraints and one on the basis of organisational regulations. Interviews were conducted with 11 GPs and as no new themes appeared in the final interviews, the sample size was not increased further. Table 1 presents a profile of the GPs in terms of their age, gender, racial group, organisational type and economic level of the community served. Two of the GPs were heading independent practitioner organisations.

**TABLE 1:** Profile of respondents.

Interviewee	Age (years)	Gender	Racial group	Organisational type	Community served
1	> 60	Male	White people	Group general practice	Middle-upper income
2	40–49	Male	Asian	Solo general practice	Lower-middle income
3	30–39	Male	Black people	Solo general practice	Lower income
4	> 60	Male	Coloured	Solo general practice	Lower income
5	> 60	Male	White people	Independent practitioner association	Not applicable
6	20–29	Female	White people	Group general practice	Middle-upper income
7	50–59	Male	White people	Group general practice	Middle-upper income
8	30–39	Male	Black people	Solo general practice	Lower income
9	40–49	Male	Coloured	Solo general practice	Lower income
10	50–59	Female	White people	Group general practice	Middle-upper income
11	40–49	Male	Asian	Independent practitioner association	Not applicable

Results are presented as themes in three broad areas: themes related to the health system and government, implementation of the NHI and the likely impact of the NHI on GP practice.

### Need for health system reform and the national health insurance

There was unanimous agreement amongst the practitioners that the NHI was needed to address the deficiencies in the current health system such as discrepancies in the distribution of resources, cost of private health care, quality of care offered by public versus private health care facilities and the need for an improved partnership between the private and public sectors. Most practitioners perceived the public sector as lacking sufficient infrastructure, being understaffed and plagued by pharmaceutical stock issues. In contrast, the private sector was reputed to give patients easier access to investigations and surgical interventions, which in turn contributed to the increased cost of private health care:

‘It would take me six weeks to get an outpatient appointment (in public) for her to go to get in to see somebody, but I could get a mammogram done this afternoon if she was in private healthcare and had a surgeon tomorrow, and I was just saying that is the kind of disparities.’ (Interview 1)

Two events seemed to have driven the escalation of private health care costs. Firstly, the competition commissioner’s banning of fees estimation by the South African Medical Association and the Board of Healthcare Funders, with the resultant loss of control over private practitioner’s fees. Secondly, government legislation on medical aids and prescribed minimum benefits for chronic conditions that had led medical aids to structure private health care costs around tertiary hospital care. General practitioners hoped that the NHI could shift the focus back to primary care and implement policies to regulate the cost of private health care:

‘I must also say that with regard to Government, that the way they’ve written the medical schemes Act and prescribed minimum benefits, they’ve basically driven private health care cost there as well. And I blame Government for that. Because what Government has done with that is allow the medical aids to structure private health care around tertiary care, and that’s why private health care is so expensive.’ (Interview 10)

### Perceptions of and relationship with government

Many practitioners voiced concerns about the government’s poor management of the current health system and that the NHI was also likely to be poorly managed and plagued by bureaucratic issues rather than effective service delivery. Appointment of health managers with political affiliations and limited health care management experience was one of the main reasons for practitioner’s low confidence in government’s ability to effectively implement the NHI:

‘I think it’s politics. I think it’s the emphasis. I think health care is suffering because there’s too much focus on politics and not on delivery.’ (Interview 10)

‘I’ve grown negative towards it. The main reason is because I see mismanagement of the current state departments, how they’re managing health, mismanaging health. So, it worries me that if its expanded into an NHI, it’s going to destroy both.’ (Interview 7)

There seems to be a strained relationship between the private sector and the government, as evidenced by antagonistic government messages. Several GPs voiced resentment that the NHI was not aimed at collaboration, but rather to eliminate the private sector:

‘There is almost nothing left for the private sector to do. Initially they said it was complimentarily and then afterwards they said no it was supplementary.’ (Interview 5)

There was also an emphasis on the government avoiding a coercive approach to enlisting GP participation in the NHI, as this was likely to result in emigration of younger GPs and poor cooperation from older GPs:

‘What I do know is that without our guys they can’t do it, that’s for sure and coercing our guys and threatening our guys will result in two things, emigration of the guys that are upwardly mobile and then you are left with a bunch of old farts who are too old to emigrate or too poor to emigrate.’ (Interview 5)

There were suggestions that the government should focus more on grassroot-level engagement with the GPs as some leaders of GP organisations and regulatory bodies were viewed as having ulterior motives and suspected of using their positions to further their own political and corporate aspirations.

### Attitude towards public–private integration of health services under the national health insurance

General practitioners felt that they had minimal input into developing NHI policy and yet their inclusion was vital for the effective implementation of affordable primary care and universal health coverage:

‘My experience as a general practitioner is that we’re not really involved in the planning and so on. The plans are being made by people in government, and what they think, and they say they’re getting input, but they don’t really get input from people that work in the areas and the regions, I don’t think they understand the quality and the input that we can give at that level.’ (Interview 10)

One of the major challenges GPs anticipated with the NHI was related to reimbursement. Some preferred an adjusted capitation payment, which considers the profile of the practice population such as age and sex, while others preferred a fee for services that was comparable to their current rates. They were sceptical about the government’s ability to make timeous reimbursements, with expectations of long delays based on their perception of current administrative inefficiencies. A previous local primary care collaboration between GPs and the government in an impoverished community ended in failure as negotiations fell apart on matters relating to reimbursement. They felt that reimbursement to GPs needed to be aligned with their current earnings and consider their practice costs. Some GPs, who were initially optimistic about joining the NHI pilot, soon became disillusioned by limited reimbursement in the pilot projects:

‘It all went along until the final point where they came with figures. And then it all fell flat, because when it came to the figures and what they’re offering, we said but you can’t offer that to us. You just can’t. And then it fell flat.’ (Interview 10)

General practitioners also voiced concerns of political differences having a significant role in the administrative inefficiencies experienced between provincial, district and municipal management teams:

‘And so, the one doctor, she gave a presentation at that meeting, and she said she really has challenges. And the challenges lie between what’s happening at provincial level, what’s happening at ward level, at municipal level, and sometimes the people don’t communicate with each other, they each have their own views, you have to work with the politicians.’ (Interview 10)

Government’s emphasis on a nurse-led primary care system under the NHI was also seen as likely to create resistance amongst GPs:

‘Dividing everybody into groups and coming out with this sort of nurse-based approach where the doctor must report to a matron and the matron is the head of the team, forget it. Doctors are not heads of team, or they don’t study for 7 years to be the second head of the team. They will never report to a matron. Never in a million years, so they have got it completely wrong. Have you ever had to report to a matron, do you know what they are like to work under?’ (Interview 5)

There were mixed feelings about the NHI pilot sites and speculation that most of the GPs that signed contracts to support public sector clinics were young doctors without a well-established practice or doctors who resigned from public sector posts to take up contracts owing to better pay:

‘And you will find that it’s usually your younger doctor that hasn’t got an established practice and went squatting somewhere and is unable to really attract, so for them it’s worthwhile seeing patients in the afternoon here and do sessions here because it sort of creates a secure income. But you will not find an established GP doing that.’ (Interview 10)

Hospitals that were joint private–public partnerships, where the private section subsidised the public section’s costs, were suggested as good role models for the NHI:

‘It was run on private grounds, where the government provided something, but the state had the responsibility, private had the responsibility to provide part of that, but equally helped to subsidise the state part. And everybody benefited. And there was accountability. When the NHI came along, I thought it’s a chance for that model to be replicated exactly like that.’ (Interview 7)

General practitioners felt that the government could also improve their engagement by offering additional benefits to offset the low reimbursement rates:

‘Potentially buying together with the state, so we can all buy whatever, tea, coffee, disposable ink cartridges, etc. Why can the state not supply at the state tender price. So, make the whole thing more attractive and give the guy R450.00 an hour. To say okay if you do that, then the following things become tax deductible. It-’s got to be a win–win not a lose–win.’ (Interview 5)

Respondents suggested that the NHI public–private collaborations could be beneficial in addressing our burden of disease by adopting a segmental approach to the NHI service delivery with government focussing on communicable diseases and delegating non-communicable disease management to the private sector.

### Implementation of the national health insurance

One of the biggest challenges, voiced by numerous GPs, related to government’s ability to implement the system and its affordability. The key areas that were highlighted included finances, accreditation, human resources, infrastructure requirements, group versus solo practices and rural health.

Policy documents were reported to lack detail regarding how the NHI would be financed and raised concerns about the sustainability of the proposed tax-based funding. The lack of continuity in leadership, following several changes of the finance minister, was deemed to have a further negative impact on the government’s ability to assess the affordability of the NHI:

‘The question, can we afford it and can we implement it, and the time frame that they are looking at. That’s my biggest challenge.’ (Interview 2)

There were fears that in order to get accredited, practices would need to incur significant additional costs, for example, by purchasing additional resuscitation equipment. There were concerns about financial restrictions and staffing requirements that would limit the number of practices that could be accredited. In view of this, policy makers should engage with GPs and discuss accreditation. General practitioners suggested outsourcing the process to private institutions that are currently involved in accreditation, such as the Council for Health Service Accreditation of Southern Africa (COHSASA):

‘But you see for instance we as GPs, we’ve been involved in accreditation process with COHSASA, everybody gave input into what we see as the standards for general practitioners. So if you want to have good quality care, go out, tell COSASA go and accredit the practices.’ (Interview 10)

The main financial issues revolved around concerns about the feasibility and affordability of the ‘big bang’ approach to NHI in the context of our country’s limited financial resources and the policy’s cost escalation. There was some support for adopting a step-wise approach and implementing the primary care phase first, which would be more affordable for the government, and then the hospital phase:

‘From a cost point, private practice is not the most expensive even for NHI. GPs get about six billion from the medical schemes so the state can afford six billion if they want to integrate.’ (Interview 8)

General practitioners felt that the private sector with its experience in managing health care would be able to provide critical input on a model for sustainable funding of the NHI. In contrast, many public sector facilities were seen as plagued by poor financial management, with no experience of being sustainable as they were always rescued by government funds:

‘Well we actually need pragmatic input, and private sector input is input from an organisation or groups that are self-funding. There is no point in actually looking to bill the system which you are not going to have to fund, because you don’t have to worry about your bottom line, which is government. So, if you don’t bring the private sector in which brings the sustaining model.’ (Interview 5)

The recruitment of nurses and doctors for the NHI was seen as a big challenge. In order to address staffing shortages, GPs opined that we need to be training more medical students and reopening nursing colleges. The recruitment of foreign doctors was likely to result in patient dissatisfaction. We also need to offer health administrator courses so that our managers have training in health issues, finance and show overall competence in being able to perform their jobs:

‘In England, they train health administrators, there’s a degree. We don’t have it here. So, you get people who are running these institutions, these administrators are really cognizant of the health issues, and they’re good at accounting and etcetera. They are people who are capable. That’s what we need, we need to train them.’ (Interview 7)

General practitioners believed that the NHI needs to have a similar approach to the UK system, whereby GPs were geographically organised into group practices and the government supported these practices, including the need for additional nursing staff:

‘What the UK did was they took all the GPs, there were the demographics, the research into the areas, they worked out, they said look okay fine, all private practices will have to come together as group practices, we will finance you, we will pay your staff, we will pay everything. We will have clinic sisters there and we will have doctors there. Come with a plan like that.’ (Interview 4)

Private practice was also seen as a base from which other health professionals or specialists could provide outreach services, creating a more comprehensive service:

‘So, your doctors, your specialists, your physios, your dietitians, optometrists, they are all governed by the Health Professions Act so they can have rooms in your area, you can have specialists to come in and then use sessional rooms. And you then establish a little centre of excellence where patients in that area have everything given to them on a plate.’ (Iinterview 5)

There was concern that many of the infrastructural and human resource challenges experienced during the pilot projects were likely to persist during the national roll-out of the NHI. A GP-centred primary care system, as currently being used by many high-income countries with a national health system, was deemed as most appropriate for the South African setting as well:

‘I’m looking at what I looked at back in the 80s, the New Zealand, Australia, Canadian and English models. They were GP centred and by and large, many of them were similar in that the government contracted with GPs to look after certain populations.’ (Interview 9)

The health practitioner shortage could be addressed by incorporating GP practices into the NHI primary care network. In metropolitan areas, GP groups like Intercare and Medicross with many GPs and allied practitioners in one facility could provide a comprehensive health care package, but in impoverished communities NHI implementation strategies need to focus on integrating solo practitioners.

They also recommended that government should provide GPs with chronic medications at state tender prices to improve pharmaceutical pricing and reduce private medical aid costs:

‘So, all medication for chronic illnesses, hypertension, diabetes, hyperlipidaemia, epilepsy, asthma, COPD. All of those coming in to a central pharmacy with little depots around the country and all patients on that, not get it through their medical aid, get it directly at state tender price, the net loser will be the pharmaceutical industry, boo hoo for them, and from that point of view, the amount that I would have to pay into a private medical aid must go down.’ (Interview 5)

An information technology (IT) platform that allowed public and private sector practitioners to access integrated patient records was seen as important. Improved communication between the two sectors would result in improved patient care and more cost-effective use of resources with less duplication of investigations and treatments. The government could learn from how private-sector organisations had developed their IT systems:

‘I want an IT platform where if I sit here I can link with the state, should be able to get access to patients I see from the state, so that I can manage them properly. But that same programme should be able to link with Discovery [private sector medical aid] as well so that I can see and just look onto what they have. And yes that would have been nice, you know if government focussed on those kinds of things, they’re going to see progress.’ (Interview 10)

The general impression amongst practitioners was that the government will be looking to engage with group practices with a multidisciplinary team within a medical centre. Some practitioners felt that the GPs aged under 50 years showed an interest in changing to group practices and investing in the latest technology and infrastructure:

‘And the under 50s are taking the approach which is group practice, automation, health care machinery, electronic. They are getting two or three practitioners, maybe a practice nurse, maybe eyeing the property next door to see, maybe we should buy that and turn it into rooms of some sort. Single switchboard instead of multiple-switchboard. Medical centre kind of feeling all under one roof, maybe a physio and an OT so long as you are all on the same register.’ (Interview 5)

However, it was deemed unwise to exclude solo practitioners as they formed the bulk of practices and played an essential role in lower socioeconomic communities:

‘We take the same scenario as the transport industry. Taxi’s started, you know the township taxi’s, they started the transport, they evolved it but when BRT [*Bus Rapid Transport*] came in, there was an attempt to take it away from the originators. The same even here. If you exclude solo practices, which is the majority of what practices are, it will be a similar parallel scenario that we will be painting. So unless you do because this was, I mean the services that are rendered, you know have been essential all along and survived with limited resources so for me you can either man that and improve on them but to try and exclude them and try to change them for other people. It wouldn’t be wise.’ (Interview 8)

Solo GP practices could link with other GPs to create virtual group practices that could provide a comprehensive care package for the community:

‘As I always say there will be a virtual practice, so there will be, say there’s five doctors in this region, so we will have a virtual practice with say 10 nurses feeding to us. Certain skills will be allocated to doctors who’ve got the skills of that, and others will be, you know, some may have a diploma in psychiatry and things.’ (Interview 10)

General practitioners felt that they needed to prepare by having an NHI training programme in place and organising funding for the required infrastructure. Poor management skills that were perceived to have been prevalent in the NHI planning process highlighted the importance of incorporating management skills into GP training programmes:

‘I mean they [*tertiary institution*] got a module training programme so that’s one of the things that to make sure that these things happen properly because whoever joins it has got proper management support. That was one of the single weaknesses within the NHI, that was actually management of the NHI. It’s very vague.’ (Interview 8)

In addressing the poor infrastructure in rural areas, GPs felt that additional clinics needed to be built and innovative ways identified to encourage urban doctors to service these rural facilities. Rural health system development requires inter-sectoral cooperation to address issues, such as road infrastructure, ambulance access, and pharmaceutical delivery systems:

‘Everybody to have access to care, which means in rural areas, they have to build clinics. They would build clinics, and then in those clinics, they have to subdivide the doctors to go to the government, for I’m not sure what, what system are they going to use to make sure that doctors are leaving the urban areas and then going and service the community in the rural areas.’ (Interview 3)

### Impact on private practice

General practitioners highlighted both negative and positive impacts that the NHI was anticipated to have on their current practice operations. Positive impacts included improved patient satisfaction, increased utilisation and income, as well as improved range of services. If the NHI provided private practices with nursing staff, then this could improve the comprehensiveness of services and include, for example, health promotion, baby clinics or vaccinations:

‘And it could be expanded, if NHI gave me a nursing sister, we could do a lot more things. We could run baby clinics, we could do vaccinations. And I would like to go into promoting health education, use my rooms to collect a group of Mommies, to talk to them about various things. Go beyond just treating disease, promoting health.’ (Interview 9)

General practitioners in less affluent areas felt that they might be forced to participate in the NHI to retain their current patients, while those in more affluent areas felt that most of their patients would still be able to afford additional private medical aid and hence their patient profile would remain unaffected. Most of the key concerns raised pertained to patient overload, inferior quality of care and increase in practice costs relating to IT infrastructure, increased staffing requirements, dispensary and accreditation. Some practitioners were hesitant to accept NHI patients as this could overload their current practice and put practitioners at risk of burn out:

‘You see, and I am going to be unhappy, because I don’t want to see more patients. I’m quite happy. That’s why I love my job still. I still love coming to work every day, because I’m not burnt out, I don’t feel like that, you know? But imagine seeing 50 patients every day. I am going to get burnt out. There’s no way you’re going to be able to provide that quality and care.’ (Interview 2)

Township practitioners felt their current patients were unlikely to be affected by the changeover to the NHI, as they would be seeing the same patients but on a different reimbursement scheme offered by the government and not by medical aids or out-of-pocket payments. They also had a more positive outlook on the NHI increasing their patient numbers, provided there was appropriate remuneration for their services:

‘I’m looking forward to NHI, because for me it’s going to be more, it’s going to be more patients. More hours, and then less money. So, we don’t want that kind of, that kind of set-up.’ (Interview 3)

There was also concern that geographically based registration of patients in the NHI would disrupt doctor–patient relationships that had developed over many years:

‘You must be happy with the doctors in your area. You know people travel kilometres to go and see their doctor. They have a relationship with them. Some of them move areas and they still come to see their doctor. Now how do you manage all of that?’ (Interview 2)

## Discussion

### Key findings

General practitioners supported the introduction of an NHI system that addresses the inequities and inefficiencies of the current health system and reduces costs of private health care. National health insurance was viewed as an opportunity to bridge the gap between the two health sectors, provided it was implemented properly. Key viewpoints of GPs are summarised in Table 2.

**TABLE 2:** General practitioners’ insights about factors that can influence their level of participation in the national health insurance.

Insight	Issues
**Perceptions of government and the NHI policy**	**Issues with implementation**
NHI is needed to tackle problems and inequity in the health system	Government lacks administrative and managerial capacity and needs to have dedicated training programmes
NHI can reprioritise primary care	Need to have a sustainable, efficient and realistic model for reimbursement that might include direct payment and other benefits
Government is antagonistic towards the private sector	Need to clarify the criteria for and implications of accreditation
Government needs to have a dialogue with GPs in terms of policy formulation and clarification	Need more infrastructure, particularly in rural areas
Coercing the private sector will lead to resistance and emigration	Roll out should be incremental, starting with primary care
GPs are negative towards being nurse-led in primary care and prefer a doctor-led approach	Group practices with multidisciplinary teams are ideal, but solo GPs need to be included
There is poor coordination between the different levels of government	Need an integrated IT system across all providers
Need to train more doctors and nurses and incentivise rural areas	Need to provide appropriate training for GPs
Government should explore models of public–private partnership and make use of private sector expertise in finances, IT and accreditation.	Younger GPs may be more likely to adapt to the NHI
GPs are concerned with political commitment to the financing of the NHI	
**Effect on practice**	
A more comprehensive service may increase patient satisfaction.	
A higher workload may decrease quality of care and increase stress or burnout.	
Higher costs to meet accreditation criteria, while effect on income uncertain.	
General practitioners in low socioeconomic areas see a more positive benefits/harm ratio.	

NHI, national health insurance; GP, general practitioner; IT, information technology.

### Discussion of findings in relation to policy and research

National health insurance aims to provide a comprehensive health care package to all South Africans through accredited public and private health care providers.^19^ Government is now moving towards the second phase of the implementation process with one of the focus areas being the design of contracts with GPs.^20^ Practitioner concerns about poor reimbursement rates, delays in payment for services and a negative impact on their current practice are key issues that need to be addressed to ensure their participation. Similar sentiments were also expressed by private practitioners in newly implemented NHI systems in other African countries, with many accredited facilities opting out of the scheme at a later stage.^21,22^ Issues relating to reimbursement delays, unfavourable reimbursement prices for medicine, extensive labour involved in processing claims and poor relationships with the governing authority featured prominently.^21^

In our setting, it could be beneficial to consider the differences between group versus solo practitioners to provide GPs with contract options based on their practice and the community’s needs. General practitioners in group practice may be more in urban areas – younger, white and female – and charge higher consultation fees than solo practitioners.^23^ Group practice GPs also have higher practice costs compared to solo practitioners, owing to their increased staffing and operational expenditure.^23^ There seems to be a perception among practitioners that the government is looking to contract mainly group practices who can offer a variety of services, which creates uncertainty amongst solo practitioners about their inclusion within the NHI. Despite this, township-based solo practitioners were more optimistic about participating in the NHI. Solo practitioners under the guidance of independent GP organisations seemed keen to create funding for their infrastructure development and training to facilitate their NHI accreditation. A previous study among private practitioners in South Africa also seemed to suggest that solo practitioners, especially in the townships, might be more amenable to contracting with the NHI.^23^

The uncertainty about GPs’ future role as primary care providers seemed to be related more with practices that would not be NHI accredited and who would be competing with the NHI service offerings. National health insurance negativity was further exacerbated by feelings that the NHI will be nurse-led, with subsequent marginalisation of the doctor’s role in primary care. However, previous research amongst South African GPs, who contracted to public clinics regularly over a long period of time, found it to be a successful private–public partnership within the community.^24^ The nursing profession, district management teams, academic institutions and independent practitioners associations all play a significant role in ensuring the success of this collaboration.^24^ Similar collaborations within the NHI could alleviate the misgivings GPs have about working together with nurse practitioners in the community.

### Limitations

This study interprets the attitudes and beliefs of GPs in Cape Town, and the findings may be transferable to similar contexts within South Africa, but cannot be seen as generalisable to all GPs. All the GPs interviewed served the Cape Town metropole area with better functioning primary care facilities than most parts of the country. This could result in some of their perspectives varying from those held by GPs in more dysfunctional health services or rural areas. General practitioners who held leadership positions were likely to have been involved in the NHI planning processes and have deeper insights about the NHI relative to the other GPs. Although the study selected a wide variety of GPs from different types of practice and serving different communities, it is possible to have missed out on GPs who might have contributed additional perspectives on the NHI.

### Recommendations and implications

Many of the concerns expressed by GPs could be tackled by greater policy dialogue and clarification, particularly in relation to accreditation and reimbursement. Dialogue with GP leadership as well as grassroots practitioners is required. A more collaborative style of engagement may help reduce fears of antagonism, coercion and marginalisation in a nurse-led system.

Ongoing research among GP organisations and GPs in different communities is required to understand how they desire to engage as the NHI evolves. Detailed exploration and piloting of provider payment methods by the NHI task team at the National Department of Health is needed to find options that will encourage GPs to participate in the NHI. Future research may be able to quantify the issues explored in this and other studies^23,25^ through a national descriptive survey of GPs.

General practitioners acknowledged the need for continuing professional development with the NHI in mind, particularly in administrative capabilities. Research on the learning needs of primary care doctors has highlighted six roles for the future GP: competent clinician; collaborator in a multidisciplinary team; change agent who improves the quality of care; critical thinker; community advocate; and capability-builder. A new national Postgraduate Diploma in Family medicine was launched in 2016 to cater to the learning needs of primary care doctors in the public and private sectors.^26^

## Conclusion

Although GPs were in agreement with the idea of NHI, there was little confidence in the government’s capability to effectively implement it and a sense of exclusion from the policy-making process. Key challenges to implementation were identified as the process of accreditation and reimbursement, and GPs were also sceptical about the managerial and administrative capacity of the public health system to implement the NHI. They identified insufficient resources, inefficient bureaucracy and poor communication as the key threats. They believed that the private sector had expertise to offer in terms of managerial capacity, sustainable financing, information technology and examples of effective private–public partnerships.

General practitioners identified potential risks to their practice as higher patient volumes, reduced quality of care and the costs of meeting the requirements for accreditation. They believed that the NHI would promote more multidisciplinary group practice and comprehensive primary care that might increase patient satisfaction. General practitioners serving lower socioeconomic populations appeared more positive but were worried that solo GPs would not be accredited. They were resistant to the idea of a nurse-led primary care service, worried about a loss of income, risk of burn out and emigration of younger doctors. They recognised the need for training to re-orientate and upskill them for additional competencies under the NHI.
